# Identification of the lymph node metastasis atlas and optimal lymph node dissection strategy in patients with resectable lung invasive mucinous adenocarcinoma: a real-world multicenter study

**DOI:** 10.1186/s40779-025-00659-3

**Published:** 2025-10-15

**Authors:** Chao Zheng, Guo-Chao Zhang, Long Zhang, Yu-Zhuo Zhang, Jia Jia, Shun Xu, Wen-Yue Zhao, Yang Liu, Meng Yue, Yue-Ping Liu, Shuang-Ping Zhang, Yi Shen, Qi-Yue Ge, Yu-Ning Han, Jing Li, Hong-Jiang Yan, Li-Yan Xue, Yu-Shun Gao, Feng-Wei Tan, Shu-Geng Gao, Qi Xue, Jie He

**Affiliations:** 1 Department of Thoracic Surgery, National Cancer Center/National Clinical Research Center for Cancer/Cancer Hospital, Chinese Academy of Medical Sciences and Peking Union Medical College, Beijing, 100021 China; 2 Department of Cancer Prevention, National Cancer Center/National Clinical Research Center for Cancer/Cancer Hospital, Chinese Academy of Medical Sciences and Peking Union Medical College, Beijing, 100021 China; 3 Department of Pathology, National Cancer Center/National Clinical Research Center for Cancer/Cancer Hospital, Chinese Academy of Medical Sciences and Peking Union Medical College, Beijing, 100021 China; 4 Department of Thoracic Surgery, the First Hospital of China Medical University, Shenyang, 110001 China; 5 Department of Radiation Oncology, Guangxi Medical University Cancer Hospital, Nanning, 530012 China; 6 Department of Pathology, the Fourth Hospital of Hebei Medical University/Tumor Hospital of Hebei Province, Shijiazhuang, 050010 China; 7 Department of Thoracic Surgery, Shanxi Province Cancer Hospital/Shanxi Hospital Affiliated to Cancer Hospital, Chinese Academy of Medical Sciences, Taiyuan, 030013 China; 8 Department of Thoracic Surgery, Jinling Hospital, Nanjing University, Nanjing, 210009 China; 9 Department of Thoracic Surgery, Ningxia Medical University General Hospital, Yinchuan, 750004 China; 10 Department of Thoracic Surgery, the Second Hospital of Hebei Medical University, Shijiazhuang, 050061 China

**Keywords:** Lung invasive mucinous adenocarcinoma (LIMA), Lymph node dissection (LND), Metastasis atlas, N-staging, Prognosis

## Abstract

**Background:**

Lung invasive mucinous adenocarcinoma (LIMA) is a rare, unique, and heterogeneous subtype of lung cancer whose patterns of lymph node (LN) metastasis are unknown, and a consensus on LN dissection (LND) has not been reached. This study aimed to evaluate LN metastasis patterns in LIMAs and establish optimal LND strategies.

**Methods:**

Data about 19,596 LNs from 1474 LIMA patients collected between January 2010 and December 2021 at 8 lung cancer research centers and tertiary hospitals across China, and data from 5304 LIMA patients between 2004 and 2021 in the SEER database were analysed. Metastasis probabilities were calculated for each LN station to construct a metastasis atlas. Statistical methods, including LOWESS fitting, restricted cubic spline, Kaplan-Meier, and logistic regression analyses, were employed to identify optimal LND strategies.

**Results:**

Compared with non-mucinous adenocarcinoma patients, LIMA patients exhibited distinct clinicopathological features and a significantly lower probability of LN metastasis (4.20% vs. 7.19%, *P* < 0.05). Metastasis was most common in the peripheral and hilar/interlobar zones (especially stations 14 and 10), with minimal involvement in the lower zone (stations 8 and 9). A U-shaped relationship between the LN count and prognosis (including overall survival, relapse-free survival, and cancer-specific survival) was found, with 6–20 and 18 LNs as the optimal range and cut-off point, respectively. Excessive or insufficient dissection was linked to poorer outcomes. A predictive model (area under the receiver operating characteristic cure = 0.8367) revealed that patients with a probability ≥ 0.5 had a significantly greater proportion of patients with stage N1+ disease (including N1 and N2 patients) (68.09% vs. 11.63%, *P* < 0.001) and worse overall survival [hazard ratio (*HR*) = 4.00, 95% CI 2.72–5.87, *P* < 0.001] and relapse-free survival (*HR* = 5.53, 95% CI 3.97–7.71, *P* < 0.001). The minimum numbers of LNs for the low- (probability < 0.1), medium- (probability 0.1–0.5), and high- (probability > 0.5) risk patients were 7, 14, and 17, respectively. For those with uncertain metastatic risk, dissecting 18 LNs may be the most appropriate and robust strategy.

**Conclusions:**

This study systematically revealed the pattern of LIMA-specific LN metastasis and proposed a risk-stratified LND strategy. These recommendations balance the imperatives of accurate staging with the preservation of long-term patient prognosis, offering a practical guideline for surgical decision-making.

**Supplementary Information:**

The online version contains supplementary material available at 10.1186/s40779-025-00659-3.

## Background

Among cancers, lung cancer accounts for the greatest number of cancer cases and related deaths in China and worldwide [1, 2]. As a rare and distinct form of non-small cell lung cancer (NSCLC), lung invasive mucinous adenocarcinoma (LIMA) accounts for only 2–5% of all lung cancer cases and is characterized by abundant mucin-producing cells and a high rate of intrapulmonary spread [3]. The previous study revealed that LIMA has a bimodal prognosis compared with that of lung non-mucinous adenocarcinoma (LNMA) [4]. Patients with early-stage disease (especially T1-2N0M0) tend to have better outcomes than LNMA patients do, whereas those with advanced disease (M1) have significantly worse outcomes [4]. Previous studies have also highlighted significant differences between LIMAs and LNMAs in terms of genetic mutations [5, 6], pathological features [7], and imaging characteristics [8]. These findings suggest that LIMA should be recognized as a unique pathological subtype, requiring personalized diagnostic and therapeutic strategies tailored to its unique biology and clinical behaviour, rather than simply applying the treatment framework used for LNMA. The strategy for lymph node dissection (LND) is a crucial component within this overall management framework.

LN metastasis is considered an early indicator of tumor dissemination, and patients with metastasis to intrapulmonary or mediastinal LNs have notably poorer prognoses than those without metastasis [9]. As an integral part of surgery for lung cancer, LND is essential for accurate pathological staging in LIMA patients and lays the groundwork for identifying candidates for adjuvant therapy [10, 11]. Conversely, LNs are also recognized as critical immune organs that play a vital role in antitumor responses [12, 13]. Some researchers suggested that excessive LND can lead to a greater incidence of postoperative complications, adversely affect the efficacy of adjuvant chemotherapy and immunotherapy, and ultimately negatively affect long-term patient outcomes [14–16]. An increasing number of studies have emphasized the importance of selective LND [17, 18].

The extent of LND should reflect an optimal balance between accurate N staging and better prognosis. Liang et al. [19] recommended 16 LNs as the optimal number for dissection in patients with resectable NSCLC. However, this recommendation does not consider specific pathological subtypes. For relatively rare LIMAs, the patterns and determinants of LN metastasis remain unclear, and no consensus has been reached on the optimal dissection strategy.

By leveraging the resources of the National Cancer Center and collaborating with multiple lung cancer research centers and tertiary hospitals across China, we aimed to comprehensively elucidate the patterns of LN metastasis in LIMA patients and develop a metastasis atlas. Furthermore, we aimed to identify the optimal number of dissected LNs for accurate N staging, establish a predictive model for assessing LN metastasis risk, and propose a tailored LND strategy for LIMA patients, thereby providing a theoretical foundation for improving patient survival and quality of life.

## Methods

### Study population

In this multicenter, real-world study, we retrospectively collected data from 1474 patients diagnosed with LIMA who underwent curative surgery between January 2010 and December 2021 at 8 lung cancer research centers and tertiary hospitals across China (Additional file 1: Table S1). All patients’ pathological tumor-node-metastasis (TNM) stages were reassessed according to the 8th edition of the American Joint Committee on Cancer Staging System [20]. Pathology reports were reviewed to determine the number of LNs harvested during surgery and the number of pathologically confirmed metastatic LNs. To verify the robustness of the results, data for 5304 LIMA patients and 59,627 LNMA patients who underwent surgery between 2004 and 2021 were retrieved from the latest version of the Surveillance, Epidemiology, and End Results (SEER) Program (https://seer.cancer.gov/) through access ID 16289-Nov2021.

This study was registered on the Medical Research platform (MR-11-23-014172, https://www.medicalresearch.org.cn) and synchronized with the Chinese Clinical Trial Registry (https://www.chictr.org.cn) platform. Ethical approval was obtained from the Ethics Committee of the Cancer Hospital, Chinese Academy of Medical Sciences (22/244-3446). Owing to its retrospective nature, the requirement for written informed consent was waived by the Ethics Committee.

### Strategies to minimize inter-center variability

To minimize the potential impact of variability across different centers, including surgical standards, pathological diagnostic criteria, and staging protocols, on the robustness of the results, uniformly standardized procedures were adopted to the greatest extent possible. Regarding surgical standards, the majority of centers involved in this study are either established regional medical centers of the National Cancer Center or have maintained long-term collaborative relationships with it, which helped foster a certain degree of consensus in surgical techniques. The National Cancer Center served as the coordinating center, implementing centralized data entry and cleaning managed by dedicated personnel. Besides, all patients’ diagnoses and staging were determined in accordance with the World Health Organization 2021 classification [3] and the 8th edition TNM staging system [20] through joint discussions between pathologists at the National Cancer Center and pathologists and/or thoracic surgeons from each participating hospital. Cases with uncertainties or disagreements were reviewed and considered for exclusion.

### Patient selection criteria

For the Chinese multicenter cohort, patients were included if they met the following criteria: 1) aged ≥ 18 years; 2) histopathologically confirmed primary LIMA; 3) received curative surgery. Patients were excluded if they: 1) had a history of malignancy within the past 3 years; 2) received neoadjuvant therapy; 3) were missing key clinical data; 4) were lost to follow-up.

For the SEER database cohort, the inclusion criteria were as follows: 1) aged ≥ 18 years; 2) histopathologically confirmed primary LIMA/LNMA; 3) underwent curative surgery. Patients were excluded if they: 1) underwent ablative surgery or had an unspecified surgical procedure; 2) received neoadjuvant radiotherapy (order of surgery and chemotherapy not provided in the SEER database); 3) were missing key clinical data; or 4) were lost to follow-up.

### Relevant concepts and definitions

To avoid confusion, it is necessary to clarify the concepts of the probability of LN metastasis and the proportion of patients with stage N1+ disease. The probability of LN metastasis refers to the ratio of metastatic LNs to the total number of LNs dissected. The proportion of patients with stage N1+ disease refers to the proportion of patients with pathologically confirmed N1 or N2 stages among the total patient population.

### Study outcomes

For the Chinese multicenter cohort, the primary endpoints were overall survival (OS) and relapse-free survival (RFS). OS was defined as the time from lung resection to death or the last follow-up. RFS was defined as the time from lung resection to relapse, metastasis, or the last follow-up. For SEER database patients, the primary endpoints were OS and cancer-specific survival (CSS). CSS was defined as the time from lung resection to tumor-specific death or the last follow-up. Survival was measured in months in all analyses.

### Construction of the LN metastasis atlas

In the Chinese multicenter cohort, patients with at least 1 LN dissected during surgery (*n* = 1332) were included to construct the LN metastasis atlas. Information on the number of LNs dissected at each station for all patients was reviewed. The LN stations and zones were defined according to the standards of the International Association for the Study of Lung Cancer (IASLC) [21]. This zoning approach is not merely a geographical aggregation but follows important anatomical boundaries within the thorax and aligns with clinical surgical dissection practices. A heatmap was used to display the metastasis status of each LN station for every individual LIMA patient and was correlated with the patient’s pathological N stage, predicted metastasis probability, and risk stratification. The total number of dissected LNs and the number of metastatic LNs at each station were recorded. The probability of LN metastasis was calculated and mapped onto an anatomical atlas. We also analysed the probability of metastasis for each LN station in patients with N1/N2 disease (*n* = 208). Within the context of precision medicine, lobe-specific LND is increasingly emphasized for lung cancer resection [22]. Therefore, we additionally explored the impact of tumor location along with other various clinical and pathological factors on the probability of metastasis for each LN station/zone.

### Statistical analysis

Continuous variables were presented as the mean ± standard deviation or median [interquartile range (IQR)]. The assumption of normality was assessed using the Shapiro-Wilk test. Differences between groups were compared via *t* tests for normally distributed data or Mann-Whitney U tests for non-normally distributed data. Categorical variables were displayed as frequencies and percentages, and differences were compared via Pearson’s Chi-square test or Fisher’s exact test, as appropriate. Specifically, post-hoc pairwise comparisons were performed with Bonferroni correction to compare the differences in metastasis rates among the 6 LN zones, with the significance level set at α = 0.0033. A histogram was generated to visually compare the distributions of patients with different numbers of dissected LNs.

Logistic regression analysis was performed to identify clinicopathological factors influencing LN metastasis. Variables with a *P*-value < 0.1 from the univariate analysis were included in the multivariate model [23]. To avoid the potential bias caused by multicollinearity, the variance inflation factor (VIF) was assessed, and a mean VIF of less than 5 was considered acceptable [24]. The final multivariate model was constructed to estimate the probability of LN metastasis. The model performance was evaluated via receiver operating characteristic (ROC) curves and area under the curve (AUC) values. To rigorously assess the model’s robustness, we randomly extracted 3 validation subsets comprising 30% (validation cohort 1), 40% (validation cohort 2), and 50% (validation cohort 3) of the Chinese multicenter cohort for ROC analysis through the createDataPartition function from the caret package. Considering that pathological features cannot be rapidly obtained during surgery, we developed a simplified prediction model by excluding pathological features from the full predictive model, and performed both internal validation (using the 3 subsets above) and external validation (using the SEER cohort). The association between mutation status and LN metastasis was analysed in patients with available genetic testing results (*n* = 322). A Sankey diagram was created to depict the relationships between patients’ predicted probability of metastasis, N stage, and the number of dissected LNs.

To determine the optimal number of dissected LNs for accurate N staging, the proportion of patients with stage N1+ disease among patients grouped according to the number of dissected LNs was calculated, and a scatter plot was generated. The curve was then fitted via locally weighted scatterplot smoothing (LOWESS) via the lowess function with default parameters, including a smoothing parameter f = 2/3 and 3 robustness iterations (iter = 3) [25]. Structural breakpoint function analysis was used to determine the optimal number of dissected LNs for prognosis. A restricted cubic spline (RCS) with 3 knots (10th, 50th, and 90th percentiles) with default parameters using the rms package was applied to assess the nonlinear relationship between the number of LNs dissected and long-term prognosis, where the curve intersection at *HR* = 1 represented the optimal number of LNs [26]. Notably, LOWESS and RCS analyses were performed based on the total number of LNs across all stations rather than selectively focusing on specific stations. This approach ensures a comprehensive assessment of the relationship between LN counts and staging accuracy or long-term prognosis. Kaplan-Meier curves were used to compare long-term survival differences among different patient groups, and differences were assessed via the log-rank test. Forest plots were employed to visualize the results of subgroup analyses, including stratification by metastasis risk and receipt of adjuvant therapy. All analyses and visualizations were performed via Stata (version 16.0 SE) and R (version 4.4.1) software. A two-sided *P*-value of < 0.05 was considered to indicate statistical significance.

## Results

### Baseline characteristics of the study cohort

In the Chinese multicenter cohort, a total of 1474 LIMA patients were included in the final analysis, comprising 1332 patients who underwent LND and 142 patients who did not (Additional file 1: Table S2). In the SEER database, 5304 LIMA patients (4794 with LND and 510 without LND) and 59,627 LNMA patients who underwent LND were included. A descriptive review of the baseline data showed that the clinicopathological characteristics of patients in the Chinese multicenter cohort were broadly consistent with those of patients in the SEER cohort (Additional file 1: Table S2). In general, for patients who underwent LND, the median numbers of dissected LNs in the Chinese multicenter cohort and the SEER database were 14 (IQR 9–19) and 9 (IQR 5–14), respectively (Additional file 1: Table S2 and Fig. S1a). The probabilities of LN metastasis were 5.29% and 4.20%, respectively, and the proportions of patients with stage N1+ disease were 15.62% and 13.22%, respectively (Additional file 1: Table S2).

### Unique LN metastasis patterns in LIMA

The clinicopathological data of 4794 LIMA patients and 59,627 LNMA patients from the SEER database were compared. Among patients with resectable tumors, compared with LNMA patients, LIMA patients had significantly larger tumors and a greater proportion of T3 and T4 tumors (Additional file 1: Fig. S1b). The number of LNs dissected in LIMA patients was slightly greater than that dissected in LNMA patients (*P* = 0.0033, Additional file 1: Fig. S1c), likely because tumor size is an important factor influencing LN metastasis, leading surgeons to increase the number of LNs dissected in LIMA patients. However, surprisingly, the probability of LN metastasis in the LIMA group was significantly lower than that in the LNMA group (4.20% vs. 7.19%, *P* < 0.05; Additional file 1: Fig. S1d). As the T stage increased, the proportions of N1 and N2 patients increased in both the LIMA and LNMA patients. However, these proportions were consistently significantly lower in LIMA patients than in LNMA patients (Additional file 1: Fig. S1e). These findings suggest that LIMA probably exhibits distinct patterns of LN metastasis.

### Construction of the LIMA-specific LN metastasis atlas

**Fig. 1:**
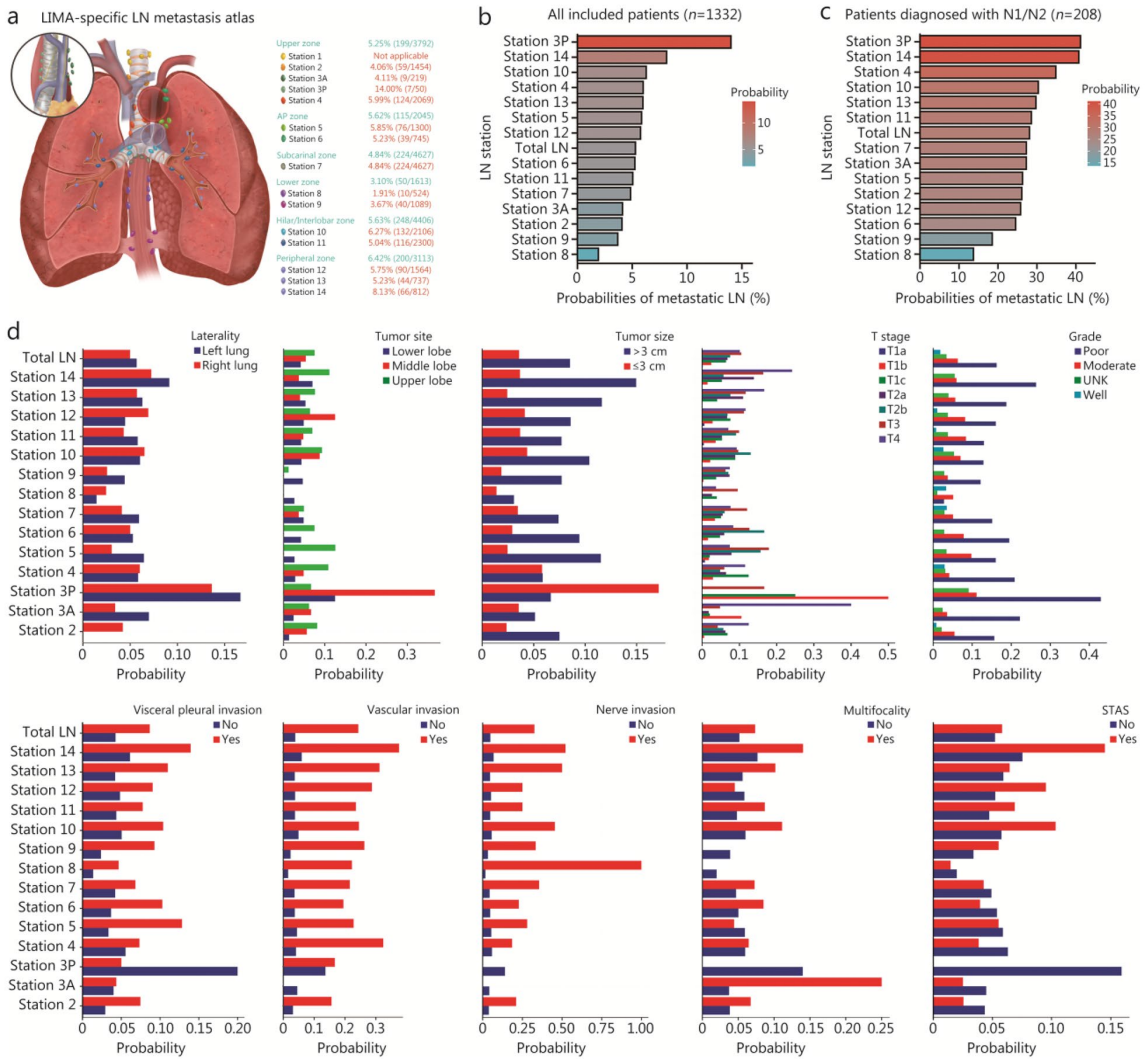
The LIMA-specific LN metastasis atlas. **a** The probabilities of LN metastasis at each station and zone in the anatomical atlas. **b** Box plots showing the probability of metastasis at each LN station in descending order for all included patients. **c** Box plots showing the probability of metastasis at each LN station in descending order for patients diagnosed with N1/N2 disease. **d** The probability of metastasis at each LN station with various clinical and pathological characteristics. LIMA lung invasive mucinous adenocarcinoma, LN lymph node, STAS spread through air spaces

A total of 19,596 LNs were dissected from 1332 LIMA patients, with 1036 pathologically confirmed as metastatic LNs. The metastasis status of each LN station for every individual LIMA patient was shown in Additional file 1: Fig. S2. Detailed information on the probability of metastasis, as well as the number of dissected and metastatic LNs for each LN station, was provided in the metastasis atlas (Fig. 1a, b). Additionally, we separately analysed the probability of metastasis in 208 patients diagnosed with N1/N2 disease (Fig. 1c). The results indicated that for all resectable LIMA patients and those diagnosed with N1/N2 disease, the highest metastasis probabilities were observed at stations 3P and 14, whereas the lowest probabilities were at stations 8 and 9. Furthermore, the impacts of various clinical and pathological factors on the probability of LN metastasis were explored. The results revealed differences between the probability of metastasis at different LN stations based on factors such as laterality, tumor site, tumor size, T stage, grade, visceral pleural invasion, vascular invasion, nerve invasion, multifocality, and spread through air spaces (STAS) (Fig. 1d). For example, patients with stage T1a disease had a nearly 0% probability of metastasis across all LN stations, whereas patients with vascular or nerve invasion had probabilities of metastasis exceeding 30% at most LN stations. In the context of growing emphasis on lobe-specific LND, it is particularly important to highlight the influence of tumor location on the metastasis probability to each LN station (Fig. 1d). Overall, patients with tumors located in the upper lobe exhibited a higher probability of metastasis compared with those with tumors in the middle lobe, followed by the lower lobe. For LN stations 2, 4, 5, 6, 10, 11, 13, and 14, tumors in the upper lobe were associated with a relatively higher metastasis probability. In contrast, for stations 3A, 3P, and 12, tumors situated in the middle lobe demonstrated the highest metastasis probability. These findings support the rationale for lobe-specific LND and suggest that relatively more extensive dissection may be warranted for upper lobe tumors.

Given the small number of dissections at specific stations, especially stations 3A and 3P, we further classified the LN stations into 6 zones according to the IASLC guidelines [21]. For all patients, the highest and lowest metastasis rates were observed in the peripheral and lower zones, respectively (Additional file 1: Fig. S3a). In patients diagnosed with N1/N2 disease, the highest and lowest metastasis rates were observed in the upper and lower zones, respectively (Additional file 1: Fig. S3b). Similarly, the probability of metastasis in different zones varied greatly depending on different laterality, tumor site, tumor size, grade, T stage, visceral pleural invasion, vascular invasion, nerve invasion, multifocality, and STAS (Additional file 1: Fig. S3c). Besides, we compared the metastasis probabilities across the 6 LN zones for the Chinese multicenter cohort. Pearson’s Chi-square test was performed, and a statistically significant difference (*χ*^2^ = 26.80, df = 5, *P* < 0.0001) across zones was found. Then, post-hoc pairwise comparisons were conducted with Bonferroni correction (significance level set at α = 0.0033). The results indicated that the metastasis probability in the lower zone (3.10%) was significantly lower than that in the upper zone (5.25%), AP zone (5.62%), hilar/interlobar zone (5.63%), and peripheral zone (6.42%) (*P* < 0.0033). Furthermore, the metastasis probability in the peripheral zone was significantly higher than that in the subcarinal zone (4.84%) (*P* < 0.0033). No statistically significant differences were observed between other zone pairs (Fig. 1a and Additional file 1: Fig. S3a).

### Relationship between the number of dissected LNs and prognosis

**Fig. 2:**
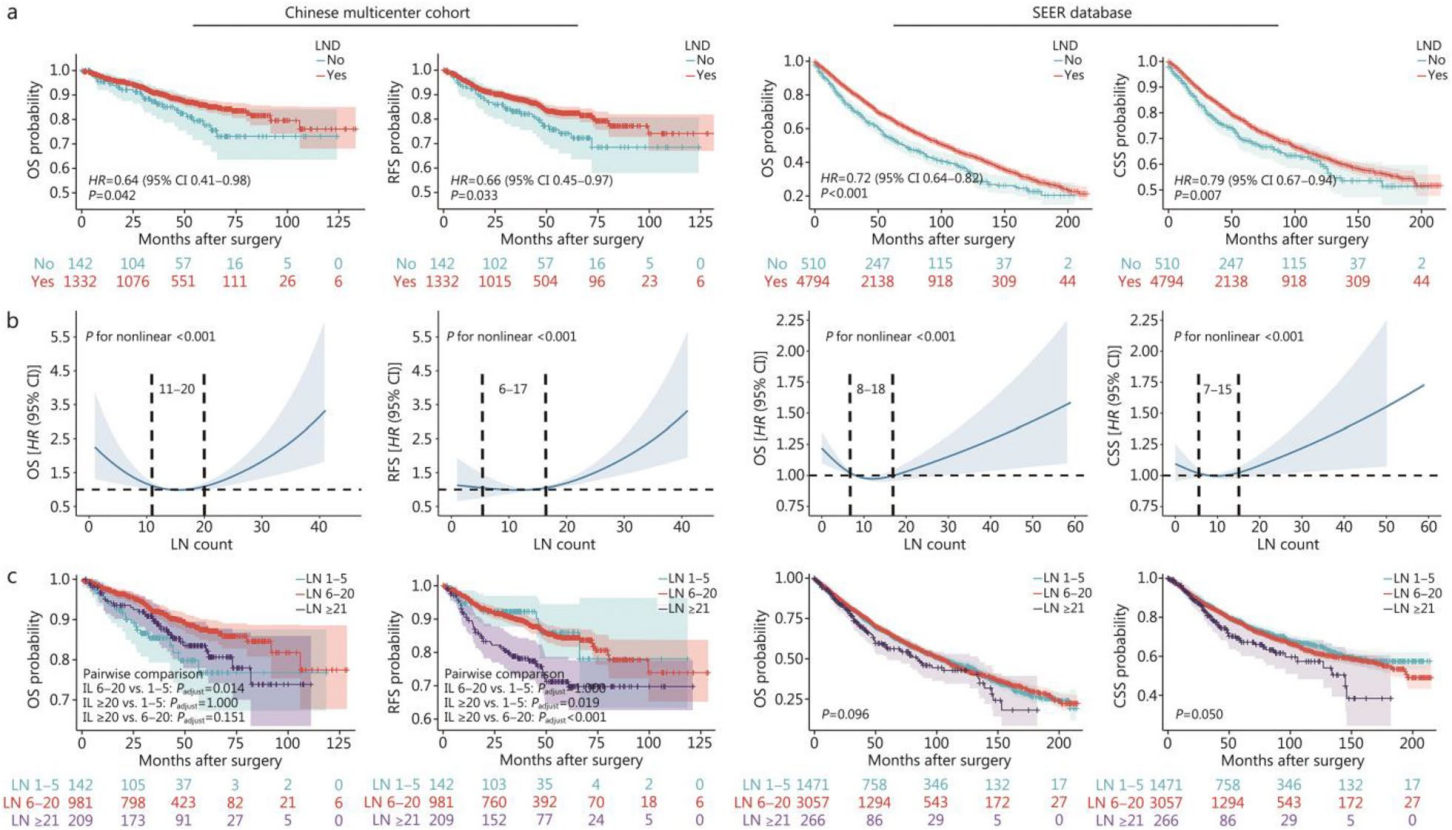
Relationship between the number of dissected LNs and patient prognosis. **a** Kaplan-Meier plot of different prognostic indicators for patients with and without LND in the Chinese multicenter cohort and the SEER cohort. **b** The restricted cubic spline curves between the LN count and different prognostic indicators in the Chinese multicenter cohort and the SEER cohort. **c** Kaplan-Meier plots between the LN count groups and different prognostic indicators in the Chinese multicenter cohort and the SEER cohort. LN lymph node, CSS cancer-specific survival, LIMA lung invasive mucinous adenocarcinoma, LND lymph node dissection, OS overall survival, RFS relapse-free survival, SEER Surveillance, Epidemiology, and End Results

For patients with resectable LIMA, those who underwent LND had better long-term prognoses than those who did not (Fig. 2a). RCS curves were used to explore the relationship between the number of dissected LNs and long-term prognosis. The results of both cohorts demonstrated a “U”-shaped curve, indicating that only an appropriate number of dissected LNs was considered reasonable and beneficial in terms of prognosis, whereas under-dissection and over-dissection were closely related to poor prognosis (Fig. 2b). The two points where the curve intersected the y-axis (*HR* = 1) were selected as the range for the number of LNs dissected. Specifically, in the Chinese multicenter cohort, the optimal ranges for dissected LNs were 11–20 for OS and 6–17 for RFS, respectively. In the SEER cohort, the optimal ranges for dissecting LNs were 8–18 for OS and 7–15 for CSS, respectively.

To identify dissection ranges associated with significantly poor prognosis while ensuring clinical practicality, we performed an integration analysis of the 4 optimal ranges derived from the Chinese multicenter cohort and SEER database for OS, RFS, and CSS. We found that the range “6–20” effectively encompassed all these intervals, serving as a highly inclusive and clinically operable common range. Opting for a single threshold, instead of retaining multiple complex thresholds, was essential to establish a simple, stable, and clinically applicable classification standard. Therefore, we determined that 6–20 was the appropriate range for LND in LIMA patients. Based on the number of dissected LNs, the patients were further divided into 3 groups: the 1–5 LNs group, the 6–20 LNs group, and the ≥ 21 LNs group. The long-term prognoses of these 3 groups were subsequently compared. The Kaplan-Meier curves showed that patients with ≤ 5 or ≥ 21 dissected LNs had relatively worse prognosis, compared with the 6–20 group, which was consistent with the results of RCS curves (Fig. 2c).

To further explore the association between dissection of more than 21 LNs and poorer prognosis, we conducted a subgroup analysis. Specifically, we separately evaluated the impact of dissection extent on survival outcomes in patients who received adjuvant therapy (*n* = 339) and those who did not (*n* = 993) (Additional file 1: Fig. S4a, b). The results demonstrated that among patients who did not undergo adjuvant therapy, extensive dissection (≥ 21 nodes) was significantly associated with worse RFS. In contrast, among those who received adjuvant therapy, no statistically significant difference in outcomes was observed. This suggests a potential interaction or confounding effect between LND extent and adjuvant treatment, which may be attributed to the distinct tumor biology of LIMA.

### Optimal LN number for resectable LIMA patients

**Fig. 3:**
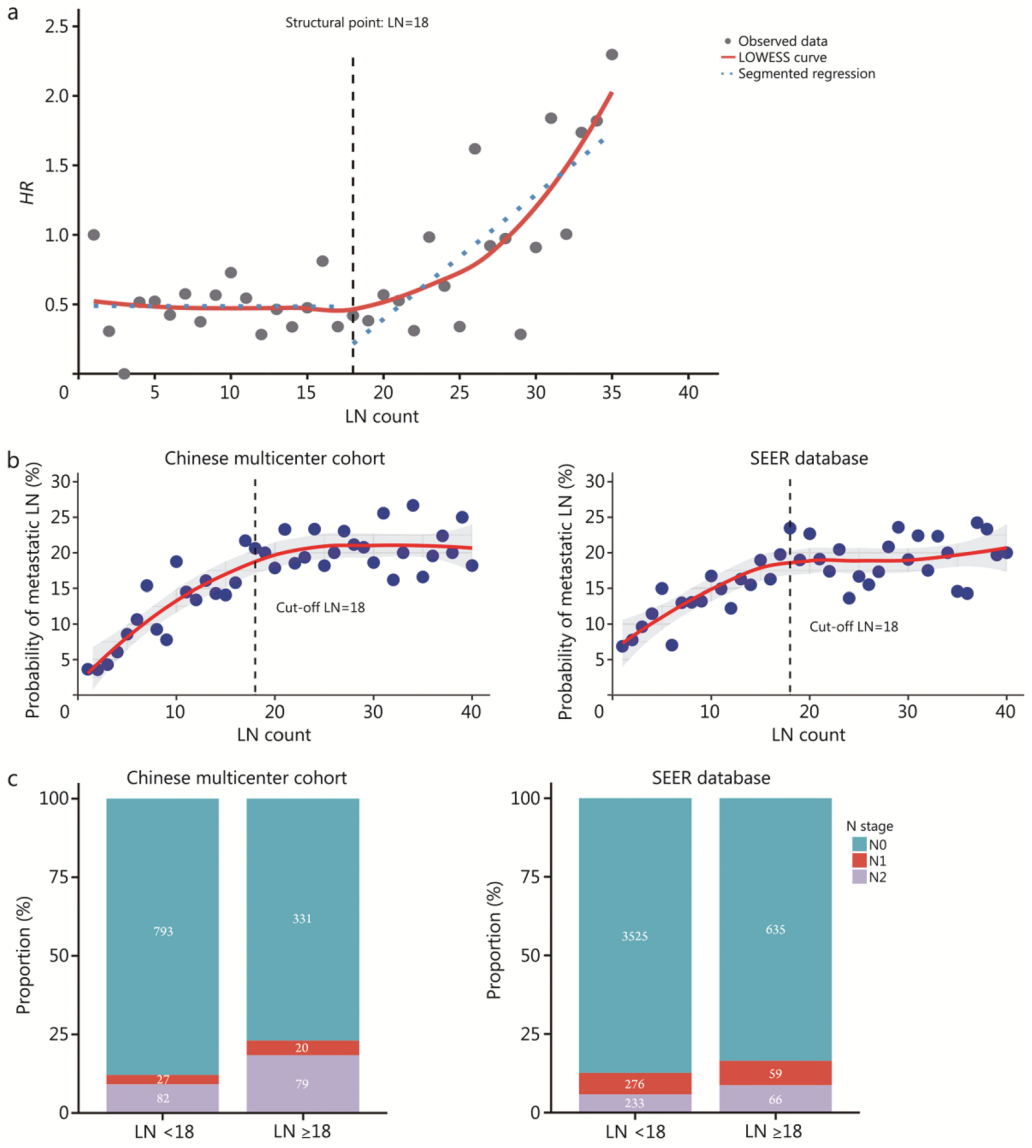
Optimal number of LNs for patients with resectable LIMA. **a** Scatter plot of LN counts and the hazard ratio of relapse-free survival for patients in the Chinese multicenter cohort. **b** LOWESS fitted scatter plots of LN counts and the proportion of patients with stage N1+ disease in the Chinese multicenter cohort and the SEER database. **c** The differences in the N stage distributions among patients with < 18 or ≥ 18 LNs dissected in the Chinese multicenter cohort and the SEER database. LIMA lung invasive mucinous adenocarcinoma, LN lymph node, LOWESS, locally weighted scatterplot smoothing, SEER Surveillance, Epidemiology, and End Results, HR hazard ratio

Considering that LND is most closely related to tumor relapse, we plotted a scatter diagram of the number of dissected LNs against RFS and performed LOWESS smoothing. Structural breakpoint function analysis was utilized to assess the presence of significant structural breakpoints. The results indicated that when the number of dissected LNs exceeded 18, the risk of relapse shifted from a stable state to an increasing trend (Fig. 3a). Therefore, the optimal number of dissected LNs for survival benefit was 18.

In addition, we analysed the optimal number of LNs for staging accuracy. The proportions of patients with stage N1+ disease (including N1 and N2 patients) with various numbers of dissected LNs were calculated, and a scatterplot with a LOWESS fit was generated. Both the Chinese multicenter cohort and the SEER database results revealed that as the number of dissected LNs increased, the proportion of patients with stage N1+ disease gradually increased until it plateaued. This analysis supported an optimal dissection number of approximately 18 LNs for staging accuracy. Beyond 18 nodes, the proportion of patients with stage N1+ disease did not significantly increase (Fig. 3b). Furthermore, when patients were grouped based on 18 LNs, the proportion was significantly greater in those with ≥ 18 nodes dissected than in those with < 18 nodes dissected (Chinese multicenter cohort: 23.02% vs. 12.08%, *P* < 0.001; SEER database: 16.45% vs. 12.62%, *P* < 0.001, Fig. 3c). Therefore, the optimal number of dissected LNs for staging accuracy was 18.

Through these two analytical methods, we determined that for patients with resectable LIMA, intraoperative dissection of 18 LNs was associated with both more accurate N staging diagnosis and better long-term prognosis.

### Personalized LND strategy based on the metastasis probability

To develop a more precise and personalized LND strategy, we constructed a probability prediction model based on various clinicopathological factors influencing LN metastasis in LIMA patients and determined the optimal number of dissected LNs for patients with different probabilities.

**Fig. 4:**
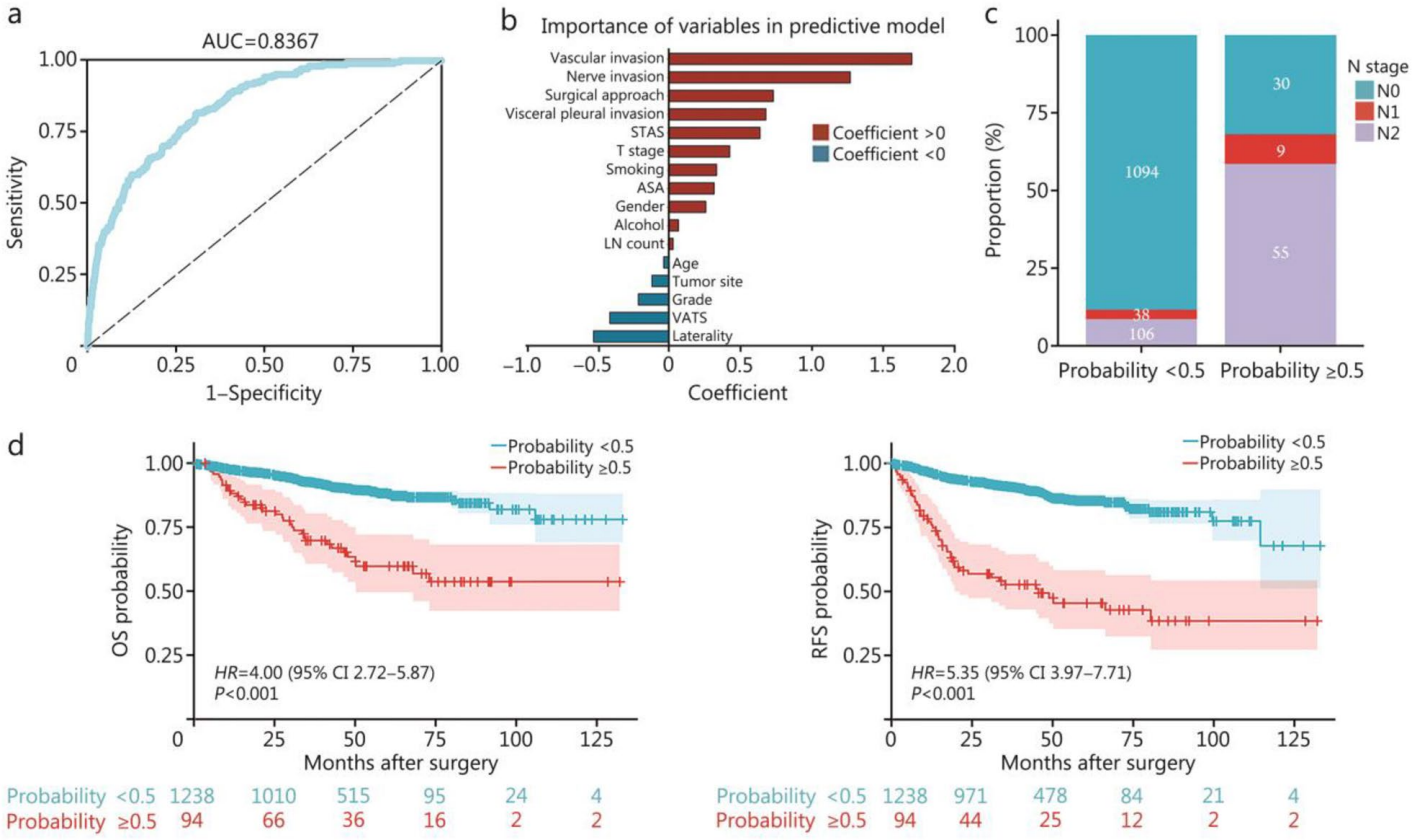
Establishment of the lymph node (LN) metastasis prediction model. **a** The receiver operating characteristic (ROC) curve of the prediction model. **b** Coefficients of variables in the predictive model. **c** Stacked bar plot demonstrating the difference in N stage distribution between the high-risk (probability ≥ 0.5) and low-risk groups (probability < 0.5). **d** Kaplan-Meier plots showing the overall survival (OS) and relapse-free survival (RFS) between the high-risk (probability ≥ 0.5) and low-risk groups (probability < 0.5). AUC area under ROC curve, CI confidence interval, HR hazard ratio

In the Chinese multicenter cohort, the proportion of patients with stage N1+ disease was 15.62%. For each patient, the presence of metastasis in any LN indicated positive N-stage disease. Therefore, we constructed a multivariate logistic regression model to calculate the estimated probability of LN metastasis (Additional file 1: Table S3). The results showed that the model had a high discriminative ability in predicting LN metastasis (AUC = 0.8367, Fig. 4a). The heatmap results revealed a high degree of consistency between the predicted probability of LN metastasis and the actual metastasis status in LIMA patients, further validating the excellent predictive performance of the model (Additional file 1: Fig. S2). The AUC values for the 3 internal validation cohorts were 0.8180, 0.8572, and 0.8371, respectively, demonstrating the stability of this predictive model across different sample sizes (Additional file 1: Fig. S5). Figure 4b showed the correlation coefficients for each variable in the model. Patients with a predicted probability of more than 0.5 had a significantly greater proportion of stage N1+ disease (68.09% vs. 11.63%, *P* < 0.001; Fig. 4c), and experienced significantly worse OS (*HR* = 4.00, 95% CI 2.72–5.87, *P* < 0.001; Fig. 4d) and RFS (*HR* = 5.53, 95% CI 3.97–7.71, *P* < 0.001; Fig. 4d) than those with a probability of < 0.5.

**Fig. 5:**
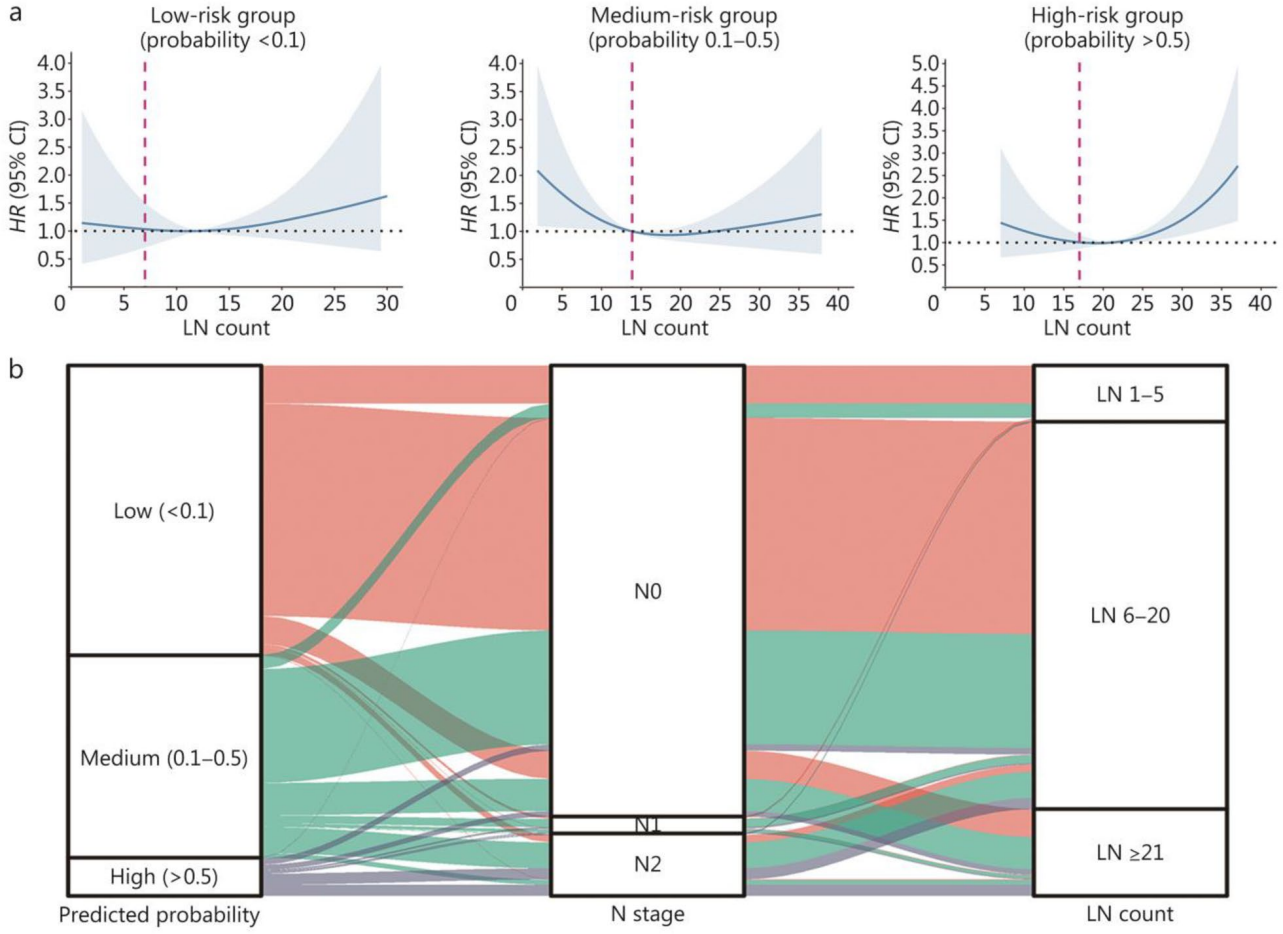
Formulation of the personalized LND strategy for patients in the low-, medium-, and high-risk groups. **a** Restricted cubic spline curve between the LN count and relapse-free survival for the low-, medium-, and high-risk groups. **b** Sankey plot showing the relationships between different risk groups, N stages, and the groups with different numbers of dissected LNs. CI confidence interval, HR hazard ratio, LN lymph node, LND lymph node dissection

Furthermore, we categorized patients into 3 groups based on their predicted probabilities: the low-risk group (probability < 0.1), the medium-risk group (probability 0.1–0.5), and the high-risk group (probability > 0.5). We plotted the RCS curves for each group and selected the first point where the curve intersected with *HR* = 1 as the minimum number of LNs to be dissected. The results indicated that the minimum numbers of LNs for the low-, medium-, and high-risk patients were 7, 14, and 17, respectively (Fig. 5a). The Sankey diagram demonstrated the relationships among the predicted probability of metastasis, N stage, and number of dissected LNs (Fig. 5b). Patients with lower predicted probabilities were mostly those with N0 disease, with most having fewer LNs dissected.

Rapid and accurate prediction of LN metastasis probability, along with the formulation of dissection strategies, is crucial for real-time intraoperative decision-making. However, the full prediction model incorporates certain pathological features that cannot be rapidly obtained from intraoperative frozen section pathology reports, which may limit its applicability in such settings. To address this issue, we endeavoured to simplify the current predictive model by performing a multivariate logistic regression analysis using only variables that can be readily acquired preoperatively and intraoperatively, excluding postoperative pathological factors (including grade, visceral pleural invasion, vascular tumor thrombus, nerve invasion, and STAS). The results demonstrated that this simplified model retained acceptable diagnostic performance, with an AUC of 0.7723 (Additional file 1: Fig. S6a), which was slightly lower than that of the full model (AUC = 0.8367). This finding indicates that the simplified model can still achieve relatively accurate predictions without relying on postoperative pathology, suggesting its promising potential for application in real-time clinical decision-making during surgery. The AUC values for the three internal validation cohorts were 0.7837, 0.7680, and 0.7663, respectively (Additional file 1: Fig. S7). Furthermore, since the vast majority of variables in this simplified model were available in the SEER database, we performed external validation in the SEER dataset. The results showed that, even in the absence of a few specific variables (including smoking, alcohol, ASA, and VATS), the model maintained considerable diagnostic efficacy (AUC = 0.7564) (Additional file 1: Fig. S6b). The detailed information of variables used in the three models was listed in the Additional file 1: Table S4.

### Association between genetic mutation status and LN metastasis

Given that the Kirsten rat sarcoma viral oncogene homologue (KRAS) mutation is a characteristic molecular feature of LIMA, exploring the relationship between mutational status and LN metastasis probability holds significant clinical relevance. Therefore, we integrated the genetic testing results from the Chinese multicenter cohort. A total of 322 patients underwent postoperative genetic testing, among whom 159 had KRAS mutations, 21 had epidermal growth factor receptor (EGFR) mutations, 10 had other alterations such as anaplastic lymphoma kinase (ALK) rearrangements, and 132 had no detectable target gene mutations (Additional file 1: Fig. S8a). We further compared the distribution of N stages and predicted probabilities of LN metastasis across different mutational subgroups. The results revealed that LIMA patients with KRAS mutations had a lower proportion of N1/N2 disease (Additional file 1: Fig. S8b) and a significantly lower predicted probability of LN metastasis (Additional file 1: Fig. S8c). This suggests a potential biological mechanism linking KRAS mutation status to LN metastasis in LIMA.

### Application of the predictive model in postoperative adjuvant therapy

Beyond guiding intraoperative decisions on the extent of LND, the predictive model also might offer significant value in postoperative management, particularly in adjuvant therapy strategies. To investigate whether the model could serve as a classifier to identify patients who might benefit from adjuvant treatment, we performed a subgroup analysis based on 3 predicted risk categories [low- (probability < 0.1), medium- (probability 0.1–0.5), and high- (probability > 0.5) risk]. In the Chinese multicenter cohort, adjuvant therapy was generally associated with poorer RFS and OS (Additional file 1: Fig. S9a, b). Forest plots revealed that adjuvant therapy was associated with worse OS in the low-risk group, and worse RFS in the low- and medium-risk groups (Additional file 1: Fig. S9a, b). In contrast, among high-risk patients, no statistically significant difference was observed in survival between those who received adjuvant therapy and those who did not.

## Discussion

The number of dissected LNs is closely linked to the accurate determination of N stage, which in turn significantly influences postoperative adjuvant therapy decision-making and long-term prognosis. Unfortunately, the optimal number of LNs to be dissected remains unclear for LIMA patients because of the relative rarity of this disease. This study reveals the unique lymphatic metastasis patterns of LIMA and presents a comprehensive and practical metastasis atlas. We identified the clinicopathological factors influencing LN metastasis and developed a predictive model with notable accuracy.

Overall, these findings suggest that both insufficient and excessive LND were significantly associated with poor prognosis in LIMA patients. The LND strategy for LIMA patients should be determined based on the probability of metastasis, required diagnostic accuracy for N staging, and long-term prognostic implications. While 18 nodes represent a robust benchmark for most resectable LIMA cases, the metastasis atlas and predictive model allow tailored adjustment of dissection extent across LN stations. Through rigorous statistical analysis, we determined that for low-, medium-, and high-metastatic-risk LIMA patients, a minimum of 7, 14, and 17 LNs should be dissected, respectively. If the metastatic risk is uncertain, we recommend that the dissection of 18 LNs is the optimal choice.

Previous studies have shown that LIMA tends to spread within the lungs, with a relatively lower rate of LN metastasis [5, 27–29]. Our findings align with this observation, as the LIMA group had a lower probability of LN metastasis and a lower proportion of patients with stage N1+ disease than the LNMA group. While constructing the LN metastasis atlas, we found that LIMA metastasis predominantly occurred in the peripheral and upper zones (particularly at stations 3P and 14), whereas the lower zone (stations 8 and 9) exhibited the least involvement, which was different from the distribution in unspecified NSCLC patients [30–32]. These findings can aid surgeons in performing more targeted LND during surgery, thereby minimizing unnecessary trauma while enhancing the removal of residual tumor. It should be noted that the results regarding LN station 3P should be interpreted with caution. In the present study, dissection of station 3P LN was performed in only 27 patients, with a total of 50 nodes retrieved. Among these, 4 patients had pathologically confirmed metastases, involving a total of 7 LNs. The limited number of patients who underwent dissection at this station may introduce bias in the probability of metastasis and the differences observed between laterality and lobes. This is likely because station 3P is not a routinely recommended dissection site according to the National Comprehensive Cancer Network (NCCN) guidelines, and its dissection in clinical practice is often closely related to the degree of LN enlargement observed on preoperative computed tomography. We look forward to future analyses with larger sample sizes to further confirm the clinical value of LN station 3P. This unique pattern of LN metastasis may be attributable to the genetic background and microenvironment of LIMA [33]. LIMA patients were reported to have a relatively high incidence of KRAS and TP53 mutations [34]. The proportion of patients with positive programmed cell death-ligand 1 (PD-L1) was much lower in the LIMA group than in the LNMA group (6.1% vs. 59.7%), whereas the proportion of patients with B7-H3 positivity was significantly greater (42.4% vs. 19.4%) [35]. This study revealed that mutational status, particularly the presence of KRAS mutation, was significantly associated with a lower probability of LN metastasis, although the underlying biological mechanism remains incompletely understood. As genetic testing has only been routinely adopted in recent years, the proportion of patients with available molecular data within the entire Chinese multicenter cohort remains relatively low, at only 24% (322/1332). Therefore, mutation status was not included in the current LN metastasis prediction model. Furthermore, multiple studies have suggested a potential correlation between the expression levels of immune checkpoints, such as PD-L1 and B7-H3, and LN metastasis [36–39]. Therefore, further investigations into the mechanisms underlying LN metastasis in LIMA patients are warranted.

This study revealed that clinicopathological factors such as vascular invasion, nerve invasion, visceral pleural invasion, and T stage were significant predictors of LN metastasis. These factors were consistently identified as critical determinants in nearly all the LN stations and zones, and they were prominent contributors to the LN metastasis prediction model, which aligns with previous findings [40]. However, some of these pathological features, such as vascular and neural invasion, are challenging to assess preoperatively. This challenge presents a strategic opportunity for advancements in precision oncology. Currently, intraoperative rapid pathology reports do not routinely include these evaluations, which limits their utility in guiding immediate surgical decision-making. Given the findings in this study, there is a compelling case for incorporating assessments of these variables into rapid pathology protocols during surgery. Tumor size and site are relatively accessible during surgery; therefore, surgeons can leverage the metastasis atlas to perform selective LND based on the specific risk profiles associated with different LN stations. To enhance the applicability of the prediction model in real-time intraoperative decision-making, a simplified version was developed. This simplified model demonstrated satisfactory predictive performance without relying on pathological features. Its generalizability was further supported through both internal and external validation.

Emerging evidence from recent studies has demonstrated that smart pathology systems powered by artificial intelligence (AI), which incorporate deep learning algorithms and computer vision technologies, can rapidly identify high-risk pathological features with accuracy comparable to that of senior pathologists [41]. Particularly for time-sensitive intraoperative decisions, such AI solutions have shown potential for reducing pathological evaluation time from hours to minutes while maintaining diagnostic precision. For instance, the study by Pan et al. [42] found that AI could rapidly identify high-risk pathological factors in patients with lung adenocarcinoma, while maintaining approximately 70% concordance with pathologists. Zhao et al. [43] found that lung cancer pathology slides could be used to rapidly identify gene mutations, with a predictive accuracy of 0.95 for the KRAS mutation. These studies suggest that the integration of AI and pathology will likely be implemented in clinical practice in the near future, enabling ultra-rapid diagnosis shortly after specimen acquisition and providing these results to clinicians for decision-making reference. Therefore, we are optimistic that the model proposed in this study holds promise for potentially assisting surgeons in intraoperative decision-making in the future.

Beyond postoperative pathological features, alternative solutions are emerging for predicting metastasis risk, such as combining preoperative imaging characteristics with ctDNA detection. Preoperative imaging is readily accessible, and with support from machine learning techniques, it can enable relatively accurate prediction. When combined with ctDNA methylation assays, this approach has the potential to reduce both false-positive and false-negative rates, thereby avoiding missed dissection of high-risk LNs and unnecessary dissection of low-risk ones. A prospective study (NCT06358222) integrating ctDNA mutation/methylation analysis with PET-CT scans was conducted for the preoperative prediction of LN metastasis status in NSCLC, which is expected to provide more alternative strategies for formulating precise LND strategies [44].

Moreover, this prediction model has significant value in postoperative management, particularly in guiding adjuvant therapy decisions and surveillance strategies. Current research indicates that, due to the unique biological characteristics of LIMA, its sensitivity to chemotherapy is relatively low, and the efficacy of radiotherapy is also limited. For unselected, resectable LIMA patients, multiple studies have shown that neither postoperative adjuvant radiotherapy nor chemotherapy significantly improved prognosis [27, 45, 46], which was similar to the result of this study. Therefore, identifying potential patients who may benefit from adjuvant therapy is of significant importance. For example, patients with low-risk might experience poor outcomes from adjuvant therapy. Avoiding unnecessary adjuvant treatment in this subgroup could help reduce therapy-related toxicity and potentially improve survival, while maintaining close monitoring for any signs of recurrence.

In addition to clinical and pathological factors, molecular biomarkers play crucial roles in predicting LN metastasis. For example, Chen et al. [47] reported that serum IGFBP7 levels were significantly higher in lung adenocarcinoma patients with LN metastasis than in those without. Dong et al. [48] discovered that macrophage-associated SPP1 was an important predictive biomarker for early LN metastasis in lung adenocarcinoma patients. Gene mutations have been identified as significant drivers of LN metastasis in lung cancer patients. For example, Guo et al. [49] reported that mutations in the ATR and TET2 genes were closely associated with LN metastasis in NSCLC patients. Similarly, Wang et al. [50] demonstrated that X7P_AMP and EGFR amplification were closely related to LN metastasis in lung adenocarcinoma patients. While these molecular factors provide valuable insights into metastatic mechanisms, the predictive model developed in the present study focused specifically on clinicopathological variables due to their immediate clinical applicability and routine availability. Future studies integrating such molecular markers with clinicopathological predictors may further enhance the accuracy of LN metastasis prediction and contribute to more personalized treatment strategies for LIMA patients.

Another significant finding is the optimal LND strategy for LIMA patients. There was a “U”-shaped relationship between the number of dissected LNs and long-term prognosis, and 7, 14, and 17 LNs were identified as the number of resections for patients with low-, medium-, and high-risk patients, respectively. This finding is clinically significant, as excessive dissection may negatively impact long-term survival, whereas insufficient dissection may increase the residual tumor burden and recurrence risk. Therefore, appropriate and moderate LND during surgery for LIMA patients is associated not only with the accuracy of N staging but also with better long-term survival. Several studies have shown that excessive LND may increase the risk of postoperative complications, thereby affecting patient recovery and long-term survival [14, 17]. Recently, Chen et al. [51] published the world’s first Phase III randomized controlled trial on selective LND in lung cancer. Focusing on patients with early-stage invasive lung adenocarcinoma presenting with ground-glass opacity-dominant lesions, the study demonstrated that when LN negativity was accurately determined preoperatively or intraoperatively, LND could be omitted. The group without LND showed no difference in survival outcomes compared to the dissection group, while also benefiting from higher surgical efficiency, fewer postoperative adverse reactions, and shorter hospital stays. Notably, follow-up data revealed a 64.5% reduction in postoperative complications, providing clinical evidence for preserving non-metastatic LNs. Zhao et al. [52] demonstrated that for clinically staged IA solid-dominant NSCLC patients, lobe-specific LND was associated with significantly fewer postoperative complications while maintaining oncological outcomes comparable to those of systematic nodal dissection. This finding was corroborated by a meta-analysis of 13 studies, which revealed that lobe-specific dissection not only decreased surgical complications but also potentially improved long-term survival [53]. Notably, Maniwa et al. [54] specifically reported that extended lymphadenectomy was correlated with increased complication rates in elderly populations.

Currently, the NCCN guidelines for NSCLC patients emphasize the importance of LN assessment (e.g., systematic LN sampling or systematic dissection), particularly station-based assessment, but the number of dissected LNs has not been established. For patients with resectable NSCLC, both N1 and N2 station LNs should be evaluated, with at least 3 N2 stations dissected [55]. With respect to mediastinal LND, for tumors in the left lung, stations 4L, 5, 6, 7, 8, and 9 are recommended for dissection, whereas for tumors in the right lung, stations 2R, 4R, 7, 8, and 9 are recommended [55].

Station-based LN assessment is not contradictory for determining the number of dissected LNs. In contrast, combining these 2 approaches can better establish individualized LND strategies. An optimal number of dissected LNs of 18 for patients with resectable LIMAs was recommended by this study to achieve a balance between complication rates and long-term prognosis. This quantitative recommendation serves as an important supplement to the current NCCN guidelines. Some studies have also made some attempts to define the optimal LN dissected. For instance, Zhao et al. [56] identified a U-shaped relationship between the number of LNs dissected and overall prognosis in all surgically treated NSCLC patients from the SEER database, including those with M1 disease. They suggested that dissecting 24–32 LNs was optimal. Liang et al. [19] proposed that for resectable NSCLC (stages I–IIIa), dissecting at least 16 LNs improved both N staging accuracy and prognosis. Recently, Zhu et al. [57] analysed 3002 early-stage NSCLC patients to determine the optimal number of LNs for dissection. They recommended that at least 10 LNs be examined for T1–3N0M0 patients, aligning with the strategy proposed in this study to appropriately reduce the extent of dissection in low-risk patients. These divergent results further underscore the distinct nature of LIMA and highlight the impact of variations in cohort composition, including tumor stage and pathologic subtype distribution.

Additionally, a comparison with other solid tumors (e.g., gastric cancer and thyroid cancer) revealed that the relationship between the number of LNs and prognosis is strongly influenced by anatomical structure and biological characteristics. For example, in gastric cancer research, the prognostic value of the LN ratio is more prominent, whereas thyroid cancer tends to dynamically adjust the scope of dissection based on imaging features [58, 59]. This suggests that the LIMA may require similar multimodal assessment optimization standards. In addition to the LN metastasis atlas and metastasis risk prediction model, exploring molecular markers for LN metastasis prediction based on multiomics data might be promising.

This study represents the most extensive investigation to date focused on LIMA patients. Notably, it represents a key contribution to the field as the systematic analysis of lymphatic metastasis patterns and proposes tailored dissection strategies for this unique NSCLC subtype. By leveraging large-scale, real-world datasets and validating the findings across multiple centers and data sources, we increased the robustness of the conclusions. Recently, the IASLC recommended incorporating both the N classification and the number or ratio of metastatic LNs for refining the N staging of lung cancer patients [60, 61]. The findings of this study provide crucial data and a theoretical foundation for developing a novel, LIMA-specific N staging system.

However, despite these strengths, several limitations should be acknowledged. First, the retrospective design of the study inherently introduced the risk of selection bias. Additionally, while uniformly standardized procedures were adopted to the greatest extent possible, data from multiple centers might also exhibit variations in diagnostic criteria, surgical techniques, and treatment decisions, which could introduce further biases. These limitations might be particularly pertinent when considering the heterogeneity of LIMA. To mitigate this bias, future studies designed with prospective data collection are needed to further validate both the predictive model and the clinical utility of its risk-stratified LND strategy. Second, the limited number of LNDs performed at specific stations, particularly stations 3A and 3P, was a challenge in this study. The small sample sizes at these stations reduced the statistical power, leading to the probabilities of metastasis that should be interpreted with caution. The low dissection rates at these stations may reflect surgical practices that prioritize other nodal zones owing to the perceived lower risk of metastasis or the technical difficulty of accessing these areas. Consequently, the findings regarding these stations, while valuable, should be validated through studies with larger cohorts and more consistent LN sampling. Third, as this was a multicenter retrospective study involving data collected from multiple hospitals, the standards and completeness of complication records (such as chylothorax, pneumonia) varied significantly across centers. Moreover, certain key complication data were missing. Consequently, we were unable to obtain complete, consistent, and high-quality complication data to perform a reliable statistical analysis.

## Conclusions

In conclusion, this study systematically reveals the patterns of lymphatic metastasis in LIMA patients and proposes an optimal LND strategy. Appropriate LND is associated not only with the accuracy of N staging but also with a better long-term prognosis to some extent. We believe that the data and models provided by this study will serve as important references for perioperative decision-making in LIMA patients, ultimately helping to improve overall survival and quality of life.

## Supplementary Information

**Additional file 1: Table S1** Distribution of 1474 patients with LIMA from eight lung cancer research centers and tertiary hospitals across China. **Table S2** Clinical and pathological characteristics for LIMA patients who received lung resection and LND in the Chinese multicenter cohort and SEER database. **Table S3** Univariate and multivariate logistic regression model for predicting LN metastasis of LIMA patients. **Table S4** Variables used in the three multivariate logistic models. **Fig. S1** Unique LN metastasis patterns in LIMA. **Fig. S2** Heatmap of the LN metastasis status of all included LIMA patients. **Fig. S3** The probability of metastasis to different LN zones. **Fig. S4** Subgroup analysis of the association between extensive LND (≥ 21 nodes) and prognosis in different adjuvant therapy status. **Fig. S5** The validation of the predictive model in three validation cohorts. **Fig. S6** Performance evaluation of the simplified prediction model for intraoperative LN metastasis prediction. **Fig. S7** The internal validation of the simplified prediction model in three validation cohorts. **Fig. S8** Association between genetic mutation status and LN metastasis in LIMA. **Fig. S9** Subgroup analysis of the association between adjuvant therapy and prognosis in different risk stratification.

## Data Availability

The datasets used and/or analysed during the current study are available from the corresponding author upon reasonable request.

## Abbreviations

ALK: Anaplastic lymphoma kinase
AI: Artificial intelligence
AUC: Area under the curve
CSS: Cancer-specific survival
EGFR: Epidermal growth factor receptor
HR: Hazard ratio
IASLC: International Association for the Study of Lung Cancer
IQR: Interquartile range
KRAS: Kirsten rat sarcoma viral oncogene homologue
LIMA: Lung invasive mucinous adenocarcinoma
LN: Lymph node
LND: Lymph node dissection
LNMA: Lung non-mucinous adenocarcinoma
LOWESS: Locally weighted scatterplot smoothing
NCCN: National Comprehensive Cancer Network
NSCLC: Non-small cell lung cancer
OS: Overall survival
PD-L1: Programmed cell death-ligand 1
RCS: Restricted cubic spline
RFS: Relapse-free survival
ROC: Receiver operating characteristic
SEER: Surveillance, Epidemiology, and End Results
STAS: Spread through air spaces
TNM: Tumor-node-metastasis
VIF: Variance inflation factor
