# Supplementary material for: Identification of the lymph node metastasis atlas and optimal lymph node dissection strategy in patients with resectable lung invasive mucinous adenocarcinoma: a real-world multicenter study
Source: Mil Med Res. 2025 Oct 15;12:67. doi: 10.1186/s40779-025-00659-3 (PMC12523043; doi:10.1186/s40779-025-00659-3)
Supplement: Supplementary file 1 — Additional file 1: Table S1 Distribution of 1474 patients with LIMA from eight lung cancer research centers and tertiary hospitals across China. Table S2 Clinical and pathological characteristics for LIMA patients who received lung resection and LND in the Chinese multicenter cohort and SEER database. Table S3 Univariate and multivariate logistic regression model for predicting LN metastasis of LIMA patients. Table S4 Variables used in the three multivariate logistic models. Fig. S1 Unique LN metastasis patterns in LIMA. Fig. S2 Heatmap of the LN metastasis status of all included LIMA patients. Fig. S3 The probability of metastasis to different LN zones. Fig. S4 Subgroup analysis of the association between extensive LND (≥ 21 nodes) and prognosis in different adjuvant therapy status. Fig. S5 The validation of the predictive model in three validation cohorts. Fig. S6 Performance evaluation of the simplified prediction model for intraoperative LN metastasis prediction. Fig. S7 The internal validation of the simplified prediction model in three validation cohorts. Fig. S8 Association between genetic mutation status and LN metastasis in LIMA. Fig. S9 Subgroup analysis of the association between adjuvant therapy and prognosis in different risk stratification. [file 40779_2025_659_MOESM1_ESM.pdf]

Table S1 Distribution of 1474 patients with LIMA from eight lung cancer research centers and tertiary hospitals across China

**Hospital**	**City, Province**	**Patient number**
National Cancer Center/National Clinical Research Center for Cancer/Cancer Hospital, Chinese Academy of Medical Sciences, and Peking Union Medical College	Beijing, Beijing	852
The First Affiliated Hospital of China Medical University	Shenyang, Liaoning	170
Guangxi Medical College Cancer Hospital	Nanning, Guangxi	112
The Fourth Hospital of Hebei Medical University/Tumor Hospital of Hebei Province	Shijiazhuang, Hebei	97
Shanxi Province Cancer Hospital/Shanxi Hospital Affiliated to Cancer Hospital, Chinese Academy of Medical Sciences	Taiyuan, Shanxi	84
Jinling Hospital, Nanjing University	Nanjing, Jiangsu	76
General Hospital of Ningxia Medical University	Yinchuan, Ningxia	73
The Second Hospital of Hebei Medical University	Shijiazhuang, Hebei	10

LIMA lung invasive mucinous adenocarcinoma

Table S2 Clinical and pathological characteristics for LIMA patients who received lung resection and LND in the Chinese multicenter cohort and SEER database

**Variables**	**Chinese multicenter cohort (*n* = 1332)**	**SEER database (*n* = 4794)**
LN count [median (IQR)]	14 (9 – 19)	9 (5 – 14)
Total LNs	19,596	50,291
Metastatic LNs	1036	2113
LN metastasis probability (%)	5.29	4.20
Gender [*n* (%)]		
Male	537 (40.32)	1978 (41.26)
Female	795 (59.68)	2816 (58.74)
Age (mean ± SD)	59.10 ± 10.29	67.86 ± 10.26
Laterality [*n* (%)]		
Left lung	618 (46.40)	1987 (41.45)
Right lung	714 (53.60)	2807 (58.55)
Site [*n* (%)]		
Upper lobe	393 (29.50)	1776 (37.05)
Middle lobe	72 (5.41)	256 (5.34)
Lower lobe	867 (65.09)	2663 (55.55)
Overlapping	0 (0)	37 (0.77)
Lung, NOS	0 (0)	62 (1.29)
Grade [*n* (%)]		
Well	96 (7.21)	2019 (42.12)
Moderate	189 (14.19)	1701 (35.48)
Poor	135 (10.14)	383 (7.99)
Unknown	912 (68.47)	691 (14.41)
Surgical approach [*n* (%)]		
Lobectomy	1158 (86.94)	3998 (83.40)
Sub-lobectomy	174 (13.06)	796 (16.60)
T stage [*n* (%)]		
T1	812 (60.96)	2491 (51.96)
T2	380 (28.53)	1226 (25.57)
T3	81 (6.08)	602 (12.56)
T4	59 (4.43)	475 (9.91)
N stage [*n* (%)]		
N0	1124 (84.38)	4160 (86.78)
N1	47 (3.53)	335 (6.99)
N2	161 (12.09)	299 (6.24)
Size [mm, median (IQR)]	22 (14 – 35)	25 (17 – 42)
Pleural invasion, present [*n* (%)]	262 (19.67)	708 (14.77)

IQR interquartile range, *LIMA* lung invasive mucinous adenocarcinoma, *LN* lymph node, *LND* lymph node dissection, *NOS* not otherwise specified, *SEER* the Surveillance, Epidemiology, and End Results Program, *SD* standard deviation

Table S3 Univariate and multivariate logistic regression model for predicting LN metastasis of LIMA patients

Variables	Univariate		Multivariate	
	***OR* (95% CI)**	***P*-value**	***OR* (95% CI)**	***P*-value**
LN count	1.05 (1.03 – 1.07)	< 0.001	1.03 (1.0 – 1.05)	0.011
Gender (male vs. female)	1.67 (1.24 – 2.25)	0.001	1.30 (0.83 – 2.03)	0.245
Age (≥ 60 years vs. < 60 years)	0.64 (0.48 – 0.87)	0.004	0.61 (0.43 – 0.87)	0.006
Age (per year)	0.97 (0.95 – 0.98)	< 0.001	-	-
Smoking (yes vs. no)	1.81 (1.32 – 2.49)	< 0.001	1.45 (0.88 – 2.39)	0.145
Alcohol (yes vs. no)	1.75 (1.21 – 2.54)	0.003	1.08 (0.65 – 1.81)	0.757
Family history of tumor (yes vs. no)	0.89 (0.61 – 1.31)	0.549	-	-
Comorbidity (yes vs. no)	0.96 (0.71 – 1.29)	0.777	-	-
Surgical approach (lobectomy vs. sub-lobectomy)	2.51 (1.40 – 4.50)	0.002	1.86 (0.95 – 3.66)	0.071
ASA classification				
II vs. I	0.27 (0.14 – 0.53)	< 0.001	0.26 (0.12 – 0.58)	0.001
III vs. I	0.57 (0.24 – 1.38)	0.217	0.32 (0.11 – 0.95)	0.040
UNK vs. I	0.42 (0.21 – 0.88)	0.014	0.77 (0.34 – 1.75)	0.537
VATS (yes vs. no)	0.35 (0.25 – 0.49)	< 0.001	0.74 (0.48 – 1.14)	0.169
Laterality (right vs. left)	0.73 (0.55 – 0.99)	0.042	0.56 (0.39 – 0.81)	0.002
Site				
Middle vs. Upper	0.68 (0.33 – 1.40)	0.297	0.91 (0.39 – 2.12)	0.835
Lower vs. Upper	0.70 (0.51 – 0.96)	0.027	0.80 (0.55 – 1.16)	0.244
Tumor size (per cm)*	1.27 (1.19 – 1.35)	< 0.001	-	-
Tumor size (> 3 cm vs. ≤ 3 cm)*	3.44 (2.53 – 4.67)	< 0.001	-	-
T stage				
T2 vs. T1	3.85 (2.75 – 5.40)	< 0.001	2.34 (1.44 – 3.81)	0.001
T3 vs. T1	3.83 (2.20 – 6.67)	< 0.001	1.88 (0.95 – 3.72)	0.068
T4 vs. T1	5.61 (3.10 – 10.16)	< 0.001	3.66 (1.77 – 7.57)	< 0.001
Grade				
Moderate vs. Well	3.14 (1.41 – 7.00)	0.005	2.70 (1.14 – 6.39)	0.024
Poor vs. Well	7.11 (3.18 – 15.85)	< 0.001	5.05 (2.14–11.94)	< 0.001
UNK vs. Well	1.43 (0.67 – 3.04)	0.350	1.02 (0.46 – 2.27)	0.963
Pleural invasion (yes vs. no)	3.41 (2.48 – 4.70)	< 0.001	1.41 (0.87 – 2.30)	0.158
Vascular tumor thrombus (yes vs. no)	10.72 (6.55 – 17.56)	< 0.001	5.50 (3.05 – 9.93)	< 0.001
Nerve invasion (yes vs. no)	17.14 (5.47 – 53.69)	< 0.001	2.34 (0.58 – 9.46)	0.233
Multifocal (yes vs. no)	1.01 (0.52 – 1.95)	0.981	-	-
STAS (yes vs. no)	2.54 (1.71 – 3.76)	< 0.001	2.28 (1.38 – 3.77)	0.001

*Tumor size was not included in the multivariate regression model due to collinearity with T stage. *ASA* American Society of Anesthesiologists, *CI* confidence interval, *LIMA* lung invasive mucinous adenocarcinoma, *LN* lymph node, *OR* odds ratio, *STAS* spread through air spaces, *UNK* unknown, *VATS* video-assisted thoracic surgery

Table S4 Variables used in the three multivariate logistic models

Variables	Full model (AUC = 0.8367)	Simplified model (AUC = 0.7723)	Simplified model using SEER cohort (AUC = 0.7564)
LN count	Yes	Yes	Yes
Gender	Yes	Yes	Yes
Age	Yes	Yes	Yes
Smoking	Yes	Yes	-
Alcohol	Yes	Yes	-
Surgical approach	Yes	Yes	Yes
ASA classification	Yes	Yes	-
VATS	Yes	Yes	-
Laterality	Yes	Yes	Yes
Site	Yes	Yes	Yes
T stage	Yes	Yes	Yes
Grade	Yes	-	-
Pleural invasion	Yes	-	-
Vascular tumor thrombus	Yes	-	-
Nerve invasion	Yes	-	-
STAS	Yes	-	-

ASA American Society of Anesthesiologists, *AUC* area under the receiver operating characteristic curve, *CI* confidence interval, *LIMA* lung invasive mucinous adenocarcinoma, *LN* lymph node, *SEER* the Surveillance, Epidemiology, and End Results Program, *STAS* spread through air spaces, *VATS* video-assisted thoracic surgery

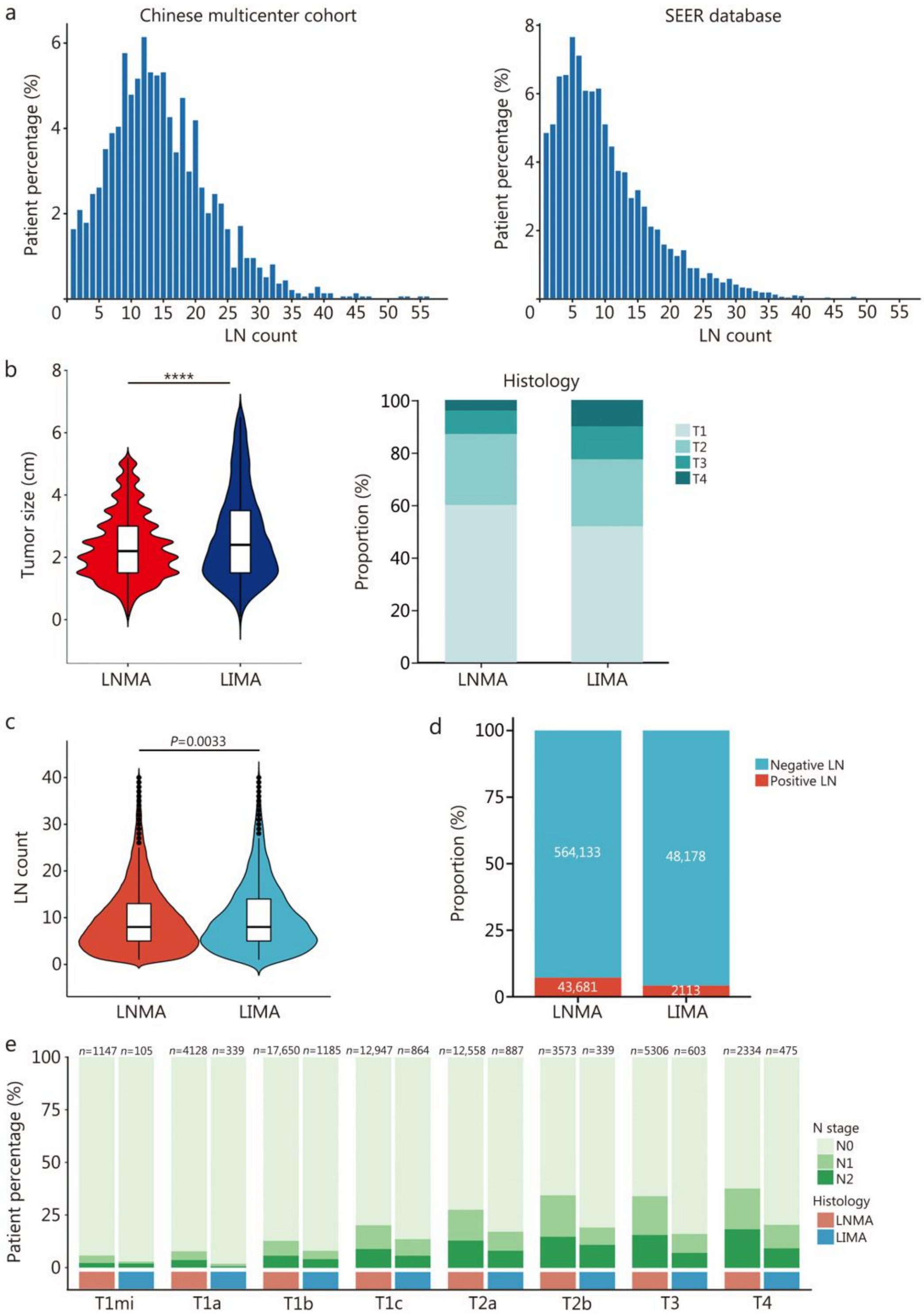

Fig. S1 Unique LN metastasis patterns in LIMA. **a** The histogram of LIMA patients with different dissected LN counts in the Chinese multicenter cohort and the SEER database. **b** Violin plot of tumour size and distribution of pathological T-stage in LIMA and LNMA patients in the SEER database. **c** Comparison of dissected LN counts between LIMA and LNMA patients in the SEER database. **d** Stacked bar plot of LN metastasis probability in LIMA and LNMA patients in the SEER database. **e** Proportion of patients with N1/N2 disease by T stage and histologic subtype in the SEER database. $^{****}P < 0.0001$. LN lymph node, LIMA lung invasive mucinous adenocarcinoma, LNMA lung non-mucinous adenocarcinoma, SEER the Surveillance, Epidemiology, and End Results Program

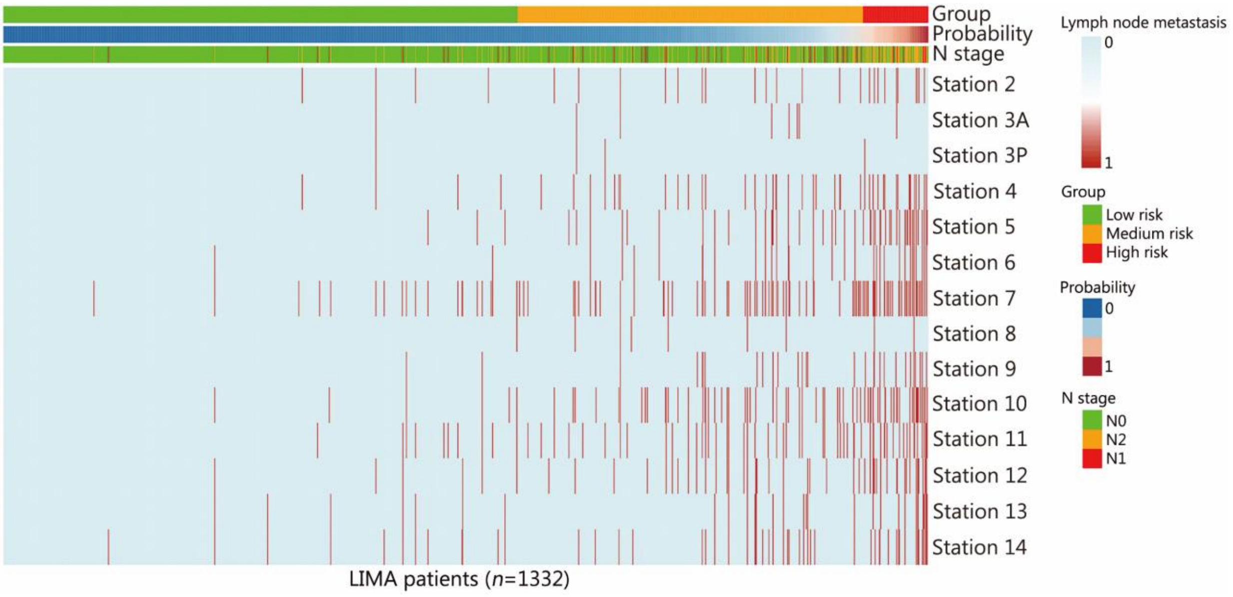

Fig. S2 Heatmap of the LN metastasis status of all included LIMA patients. LN lymph node, LIMA lung invasive mucinous adenocarcinoma

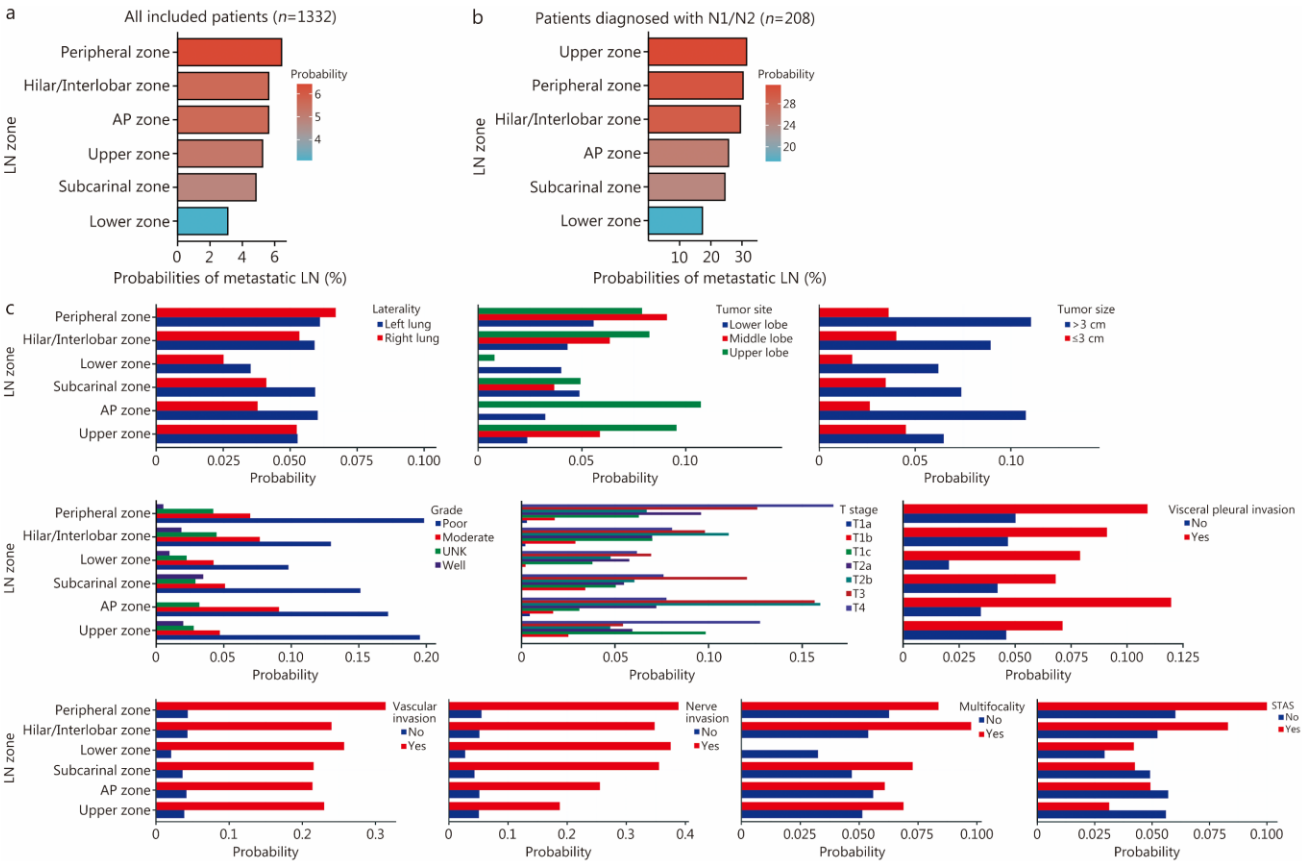

Fig. S3 The probability of metastasis to different LN zones. **a** Distribution of metastatic probability for each LN zone (in descending order) among all included patients. **b** Distribution of metastatic probability for each LN zone among patients diagnosed with N1/N2 disease. **c** The probability of metastasis at each LN zone with various clinical and pathological characteristics. LN lymph node, STAS spread through air spaces

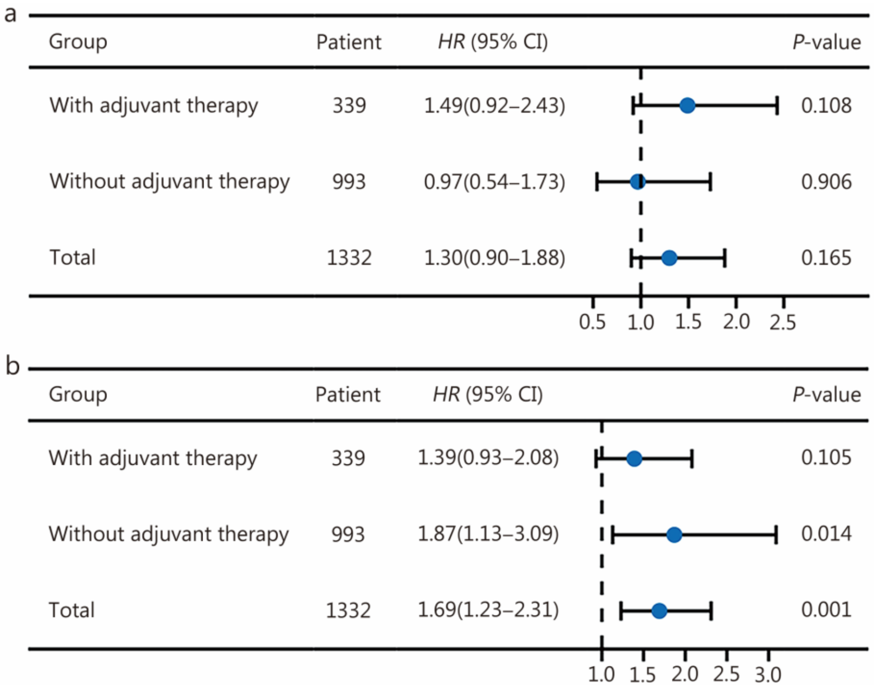

Fig. S4 Subgroup analysis of the association between extensive LND (≥ 21 nodes) and prognosis in different adjuvant therapy status. **a** OS. **b** RFS. CI confidence interval, HR hazard ratio, RFS relapse-free survival, OS overall survival, LND lymph node dissection

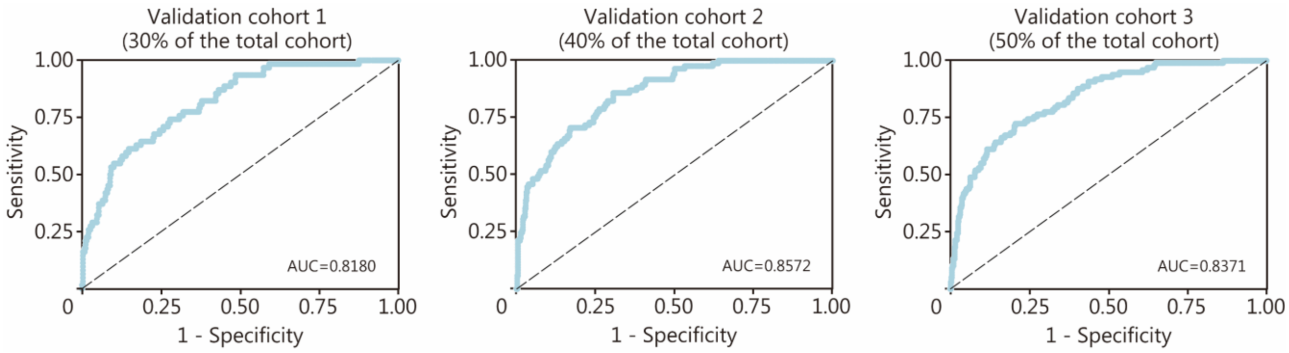

Fig. S5 The validation of the predictive model in three validation cohorts. ROC receiver operating characteristic. AUC area under the ROC curve

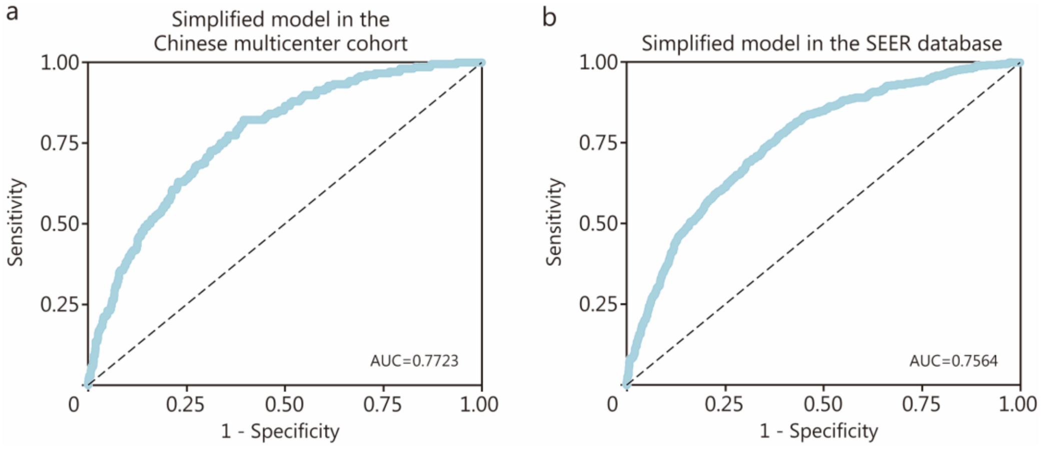

Fig. S6 Performance evaluation of the simplified prediction model for intraoperative LN metastasis prediction. **a** ROC curve of the simplified model in the Chinese multicenter cohort. **b** ROC curve of the simplified model in the external validation cohort from the SEER database. LN lymph node, ROC receiver operating characteristic, AUC area under the ROC curve, SEER the Surveillance, Epidemiology, and End Results Program

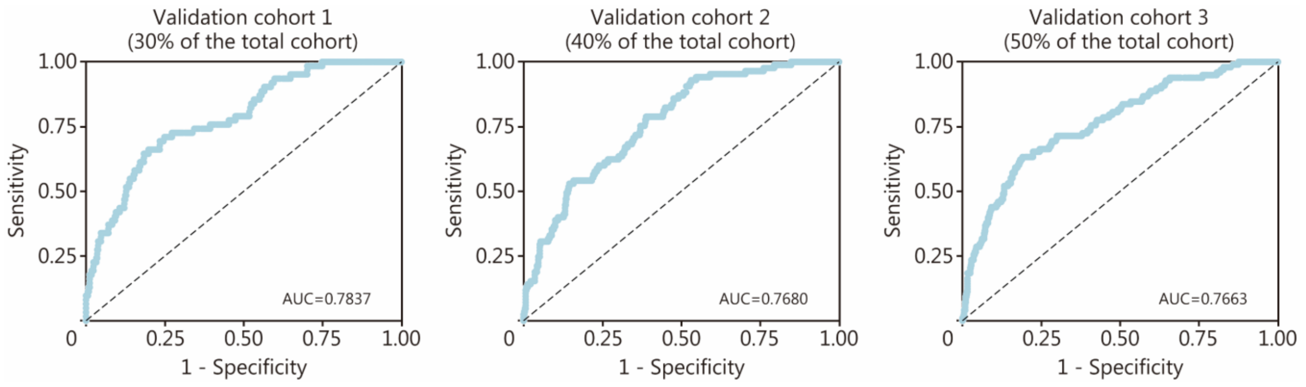

Fig. S7 The internal validation of the simplified prediction model in three validation cohorts. ROC receiver operating characteristic. AUC area under the ROC curve

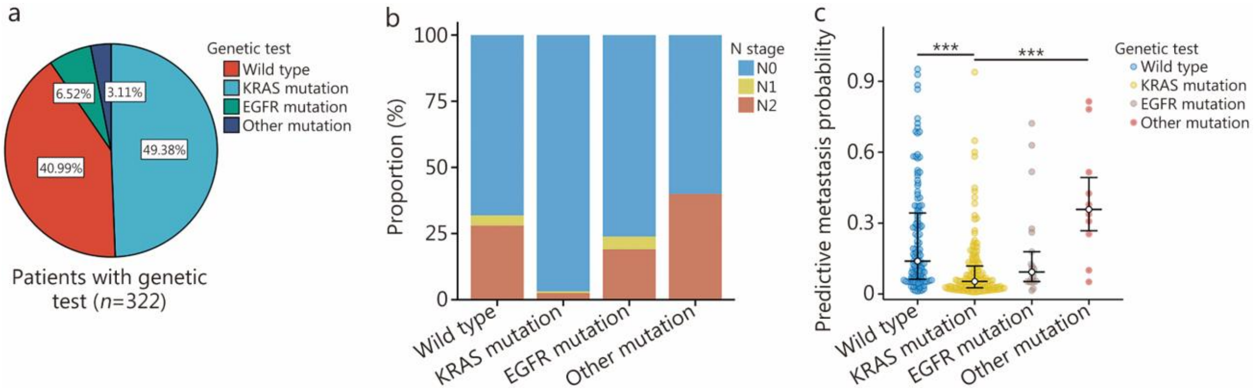

Fig. S8 Association between genetic mutation status and LN metastasis in LIMA. **a** Distribution of detected gene mutation among 322 patients who underwent postoperative genetic testing. **b** Proportion of patients with N staging across different mutational subgroups. **c** Predicted probability of LN metastasis using the full predictive model in patients stratified by gene mutation status. LN lymph node, EGFR epidermal growth factor receptor, KRAS Kirsten rat sarcoma viral oncogene homologue

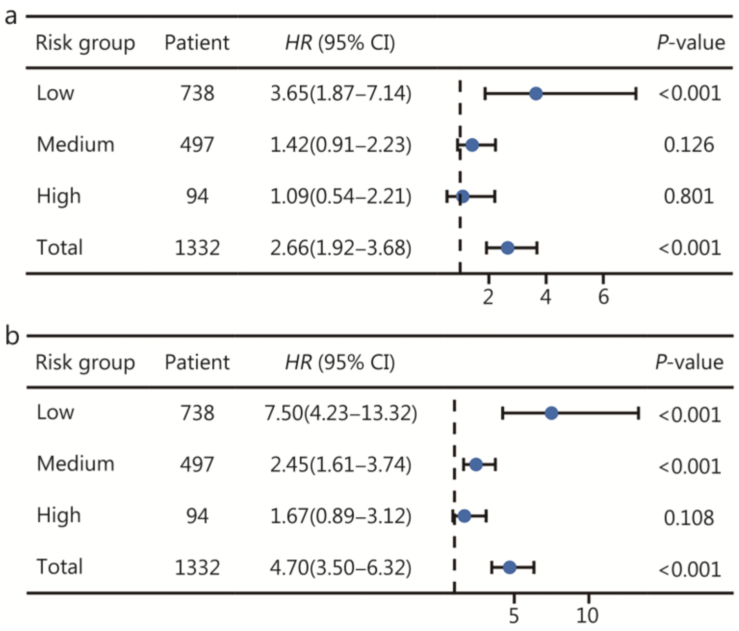

Fig. S9 Subgroup analysis of the association between adjuvant therapy and prognosis in different risk stratification. **a** OS. **b** RFS. CI confidence interval, HR hazard ratio, RFS relapse-free survival, OS overall survival
